# Recent Updates on Multifunctional Nanomaterials as Antipathogens in Humans and Livestock: Classification, Application, Mode of Action, and Challenges

**DOI:** 10.3390/molecules28227674

**Published:** 2023-11-20

**Authors:** Samreen Sadiq, Iltaf Khan, Zhenyu Shen, Mengdong Wang, Tao Xu, Sohail Khan, Xuemin Zhou, Ali Bahadur, Madiha Rafiq, Sumreen Sohail, Ping Wu

**Affiliations:** 1 School of Biotechnology, Jiangsu University of Science and Technology, Zhenjiang 212100, China; samreenkhan@stu.just.edu.cn (S.S.); 17788362661@163.com (Z.S.); mengdong@cau.edu.cn (M.W.); xu839597748961213@163.com (T.X.);; 2 School of Environmental & Chemical Engineering, Jiangsu University of Science and Technology, Zhenjiang 212100, China; doctoriltafkhan@just.edu.cn; 3 Department of Pharmacy, University of Swabi, Khyber Pakhtunkhwa 94640, Pakistan; pharmacistsohail11@gmail.com; 4 College of Science, Mathematics, and Technology, Wenzhou-Kean University, Wenzhou 325060, China; alibahadur138@gmail.com; 5 Key Laboratory for Preparation and Application of Ordered Structural Materials of Guangdong Province, Department of Chemistry, Shantou University, Shantou 515063, China; 6 Department of Information Technology, Careerera, Beltsville, MD 20705, USA; sumreensohail1994@gmail.com

**Keywords:** multifunctional nanomaterials, antipathogen application, mechanism, challenges

## Abstract

Pathogens cause infections and millions of deaths globally, while antipathogens are drugs or treatments designed to combat them. To date, multifunctional nanomaterials (NMs), such as organic, inorganic, and nanocomposites, have attracted significant attention by transforming antipathogen livelihoods. They are very small in size so can quickly pass through the walls of bacterial, fungal, or parasitic cells and viral particles to perform their antipathogenic activity. They are more reactive and have a high band gap, making them more effective than traditional medications. Moreover, due to some pathogen’s resistance to currently available medications, the antipathogen performance of NMs is becoming crucial. Additionally, due to their prospective properties and administration methods, NMs are eventually chosen for cutting-edge applications and therapies, including drug administration and diagnostic tools for antipathogens. Herein, NMs have significant characteristics that can facilitate identifying and eliminating pathogens in real-time. This mini-review analyzes multifunctional NMs as antimicrobial tools and investigates their mode of action. We also discussed the challenges that need to be solved for the utilization of NMs as antipathogens.

## 1. Introduction

Pathogens are microorganisms, including bacteria, viruses, fungi, and parasites, that may induce acute and chronic infections in their hosts after entering the body through ingestion, inhalation, or direct contact. These pathogens are directly responsible for millions of annual cases of infections and fatalities worldwide [1]. Nanomaterials (NMs) with dimensions under 100 nm offer unique characteristics that are suitable for various applications [2]. Currently, NMs are attracting significant attention, transforming antipathogen livelihoods. NMs can quickly enter bacterial, fungal, and protozoal cell walls and viral particles to perform their antipathogenic activity, owing to their ultra-small size, improved surface-to-volume ratio, greater reactivity, and high band gap [3,4]. Thus, researchers are designing nanostructures with several antipathogenic advantages. Ultimately, silver NPs (Ag-NPs) were among the first NPs with remarkable antipathogenic effects [5]. Transition metals (Ag, Cu, Zn), metal oxides (Fe_2_O_3_, TiO_2_, ZnO_2_), and carbon-based NMs also have intrinsic antipathogenic effects [6]. Furthermore, because of some pathogen’s resistance to currently available medications, the antibacterial, antiviral, antifungal, and antiparasitic performances of NMs are becoming important [7,8].

Hence, NMs offer the potential for developing new therapeutic strategies, such as drug administration and diagnostic tools for antipathogens [9]. Herein, NMs have significant characteristics that facilitate the identification of pathogens and their elimination in real time. For example, the utilization of NMs as drug carriers is an efficient way to fight against several pathogens [10,11]. NMs as a novel drug delivery method allow drugs to cross cell membranes and enter the cytoplasm, effectively killing intracellular infections and improving drug effectiveness against pathogens while minimizing negative consequences in humans and animals [12,13]. In brief, NMs, with their antimicrobial properties, are promising for emerging applications, but their function, structural characteristics, and therapeutic effectiveness remain unexplored, necessitating additional research for optimal execution [14].

This mini review aims to provide a detailed analysis of the classification, production, characterization, mode of action, and outcomes of NMs as antimicrobial agents. The main objective of this study is to explore the potential of NMs in overcoming drug resistance and enhancing the effectiveness of therapeutic interventions. In a more comprehensive approach, our study delved into the considerations pertaining to the long-term sustainability and adaptability of NMs across various environments. This review also provides insights and perspectives that will contribute to the understanding of the pathogens proliferation mechanism as well as the potential for expanding nanotechnology-based techniques for combating pathogens.

## 2. Overview of Nanomaterials

### 2.1. Classification of Nanomaterials

The classification of NMs is diverse. Based on their spatial features on the nanometer scale, NMs can be divided into zero-dimensional NMs, one-dimensional NMs, two-dimensional NMs, and nanostructured materials. Based on their morphology, NMs can be classified into nano powder materials, nano-bulk materials, nanofiber materials, nanofilm materials, and nano-liquid materials [15]. Based on their function, NMs can be categorized into nano-magnetic materials, nano-biomaterials, nano-pharmaceutical materials, nano-catalytic materials, nano-wave-absorbing materials, and so on. Based on their chemical composition, NMs can be divided into organic NMs, inorganic NMs, and nanocomposites [16,17].

#### 2.1.1. Organic Nanomaterials

Organic NMs are carbon-based compounds with covalent bonds, which provide mechanical strength, electrical conductivity, and thermal stability. They are suitable for various applications, such as portable electronics and medication delivery systems. Some examples are carbon nanotubes (CNTs), graphene, polymer NPs, and lipid-based NPs for gene therapy [18,19,20].

#### 2.1.2. Inorganic Nanomaterials

Inorganic NMs are non-carbon-based elements with unique physiochemical characteristics that are used in various domains. Metals like Au, Ag, iron (Fe), and platinum (Pt) are used in medication delivery, imaging, and biosensors. Metal oxide NMs (TiO_2_, Fe_3_O_4_, and ZnO) are used in photocatalysis, while QDs and semiconductor NMs have size-dependent optical features for next-generation technologies and photovoltaic panels [19,21,22].

#### 2.1.3. Nanocomposites

Nanocomposite, also known as hybrid NMs that refers to the combination of two or more distinct components such as polymer, metallic, or ceramic nanomaterials. These nanocomposites are used in numerous fields, such as automobiles, aerospace, and architecture. For example, Mg/CNT offers a higher tensile strength, fatigue resistance, and wear resistance, while thermoplastic/thermoset nanocomposites offer easy recycling, longevity, and chemical resistance. The Al_2_O_3_/SiO_2_ nanocomposite is a popular electrical insulator in electronics, aviation, and cars, superior to conventional polymer composites in the electric and healthcare sectors [23,24,25].

### 2.2. Synthesis and Characterization of Nanomaterials

NMs are generally manufactured through two different approaches: top-down and bottom-up methods. Top-down methods involve applying physical, chemical, or mechanical treatments to reduce large-scale materials to the nanoscale level [26,27]. Examples of top-down methods are ball milling, plasma arc synthesis, and lithography techniques. On the other hand, in the bottom-up process, NMs are synthesized from smaller building blocks like atoms, molecules, or NPs; examples of bottom-up methods are chemical synthesis, sol–gel synthesis, and vapor phase deposition approaches [28,29]. Material characterization helps in the design of new materials by understanding their chemical, mechanical, physical, and microstructural properties. Tools for characterization, include structural characterization, surface characterization, elemental analysis, surface charge characterization, crystallinity, pore structure characterization, and thermal stability. Moreover, Fourier transform infrared spectroscopy (FT-IR), nuclear magnetic resonance (NMR), UV-Vis spectroscopy, transmission electron microscopy (TEM), scanning electron microscopy (SEM), X-ray photoelectron spectroscopy (XPS), and X-ray crystallography (XRD) are the most popular techniques to improve advanced, cutting-edge materials [20,30,31].

### 2.3. Multiple Functions of Nanomaterials

**Figure 1:**
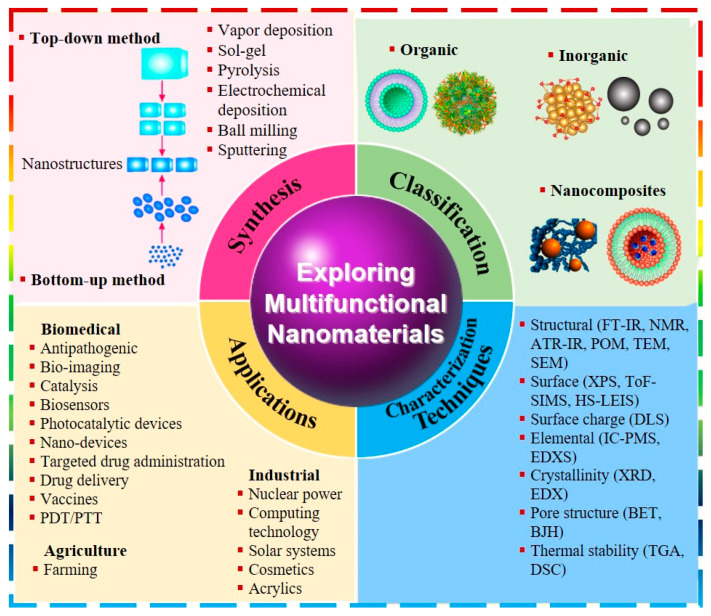
Diagrammatic illustration depicting synthesis, classification, characterization techniques, and applications of multifunctional nanomaterials.

Significantly, NMs possess remarkable characteristics including their surface area, porosity, pore volume, biocompatibility, non-toxicity, electromagnetic properties, and biodegradability. These properties enable innovation in various domains, including biomedical, agriculture, and industrial applications [32]. For example, NM-based diagnostic systems offer high specificity, low detection limits, and portability, making them valuable tools for pathogen detection and surveillance [33]. They revolutionize medicine by enabling targeted drug delivery, regenerative medicine, and biosensors [34]. NMs are known to improve livestock efficiency, carcass characteristics, intestinal microbiota, and prevent oxidative harm due to their growth-promoting, immune-stimulating, and antimicrobial properties when added to animal feed [35]. In addition, NMs are essential for energy technologies, environmental remediation, electronics, and solar system manufacturing, enabling miniaturization, higher computing power, and enhanced device performance [36,37,38]. In short, NMs are expected to play a significant role in fostering future advancements as shown in Figure 1.

### 2.4. Role of Nanomaterials as Delivery Systems That Enhance the Antimicrobial Activity of Potential Agents

Nanomaterials hold great promise in drug delivery systems, biomedicine, and environmental protection due to their unique properties such as high surface area, large pore volume, high porosity, and quantum effects. By establishing the optimal storage and delivery conditions, they can significantly enhance the efficacy of antibacterial medicines. Nanomaterials can encapsulate antimicrobial agents, enabling them to bypass cell walls, enter cells, or attach directly to microbes. Their magnetic responsiveness and photothermal properties regulate medication release, increase drug stability, and enhance efficacy. However, nanoparticles may have harmful effects on cells and microbes, and their interaction may alter their pharmacokinetic features [39]

## 3. Research Advances of Nanomaterials as Antipathogens

Recently, NMs have gained significant attention for their antimicrobial properties, which have been shown to be effective in combating various pathogen-related diseases, including those caused by bacteria, viruses, fungi, and parasites. These NMS provide enhanced antimicrobial activity, reduced antibiotic resistance, controlled drug delivery, surface disinfection, and rapid diagnostics [40]. Summary of nanomaterials for antimicrobial activities is given below in Table 1.

### 3.1. Nanomaterials for Antibacterial Applications

#### 3.1.1. Organic NMs

NMs are a promising method for combating bacteria and resistant microbes, offering antibacterial properties in organic, inorganic, and hybrid forms. NMs diverse chemical structures, particularly nanosized ones, are designed to combat highly antibiotic-resistant biofilms. Moreover, NMs have improved antimicrobial medicine’s effectiveness by dissolving and dispersing biofilms, which pose a significant barrier in clinical settings. For example, Liu and colleagues [41] demonstrated polymeric micelles as nanocarriers for hydrophobic antimicrobials such as Triclosan. Triclosan is a pH-responsive shell that targets *S. aureus* biofilms, allowing bacterial lipases to break down biofilms and release encapsulated medicines. Antibiotic-resistant bacteria demand innovative antibacterial medications, with organic NMs gaining attention for their tailored size and large surface-to-volume ratio [101]. In contrast, Costa et al. [102] explored rifampicin-containing poly-lactic acid (PLA) NPs functionalized with poly-L-lysine (PLL), which reversed negative charges to improve antibiotic delivery in *S. aureus* biofilms, enhancing carrier retention capacity and treatment efficacy. Moreover, Hoque and colleagues [42] found that N-(2-hydroxypropyl)-3-trimethylammonium chitosan chlorides effectively kill MDR bacteria by disrupting the bacterial membrane and exhibiting minimal resistance. In vitro, results confirmed their non-toxic behavior, low skin tissue inflammation, and reduced methicillin-resistant *S. aureus* (MRSA) burden in superficial skin infections without adverse effects.

In addition, nanotechnology and NMs have significantly impacted the field of livestock antibacterial medicine [43]. Multiple studies have shown that ampicillin-loaded chitosan NPs can suppress *E. coli* growth, prolong ampicillin release, and improve animal welfare. Liposomes, an amphiphilic delivery system, enhance meat preservation by encapsulating active compounds, extending shelf life, and promoting antibacterial and antioxidant effects [103]. For instance, Singh and coworkers [44] demonstrated that liposomes and solid-lipid NPs can improve meat ingredient surface quality, effectiveness, stability, sensory quality, and bioavailability, while essential oil nano emulsions offer antibacterial and antioxidant properties. Recently, Li and colleagues [45] discovered that administering liposome-associated fimbriae antigens to chickens at 8 and 10 weeks of age increased their IgA and IgG responses and reduced *Salmonella enterica* excretion. Additionally, researchers have developed NPs for enhanced feed detection in poultry. They have developed a nanomaterial-based technology for detecting nanoparticles in poultry feed. This technology can improve the sensitivity and accuracy of detection, resulting in better control of feed quality and safety. NPs with a polystyrene base, PEG linker, and mannose-attracting biomolecule could potentially replace antibiotics and reduce antibiotic-resistant bacteria [104].

#### 3.1.2. Inorganic NMs

Inorganic NMs exhibit enhanced antibacterial activity, biosensing, broad spectrum, and drug delivery capabilities against both Gram-positive and Gram-negative bacteria [105]. Biosensors use enzymes or antibodies to assess the effectiveness of sterile agents, enabling real-time detection of drug-resistant strains for effective therapies and infection control. Accordingly, Fouda and his research team [46] discovered selenium NPs (Se-NPs) as versatile therapeutic agents, biocides, antioxidants, catalysts, and photoreactive substances. They offer broad-spectrum defense against bacteria, cancer, fungi, and pathogens, exhibit photocatalytic performance, and can be recycled five times. Additionally, inorganic NMs are photothermal candidates that convert light energy into heat to target bacteria, killing germs without destroying healthy tissues [106]. Chen and colleagues [47] found that Y-4-produced palladium NPs have broad NIR absorption, making microorganism eradication easy, economical, and sustainable against Bacillus megaterium.These NPs improve dispersity, light utilization stability, biocompatibility, and photothermal efficacy against *S. aureus* and *E. coli*.

In addition, Adegbeye and coworkers [107] demonstrated that inorganic NPs like Ag and Cu can improve feed efficiency, prevent periodontal disease in horses, and address issues like environmental pollution, antibiotic resistance, digestive disorders, and gut health management. Additionally, ZnO-NPs have the potential to be used as antibiotic and anticoccidial replacements due to their bioavailability, characteristics, and impact on veterinary biological systems [108]. In this regard, Yusof and his team [48] highlighted the effectiveness of ZnO-NPs as an alternative antibiotic against multidrug-resistant bacteria in the poultry industry, inhibiting the growth of *Salmonella* spp., *E. coli*, and *Staphylococcus aureus*. Moreover, Hasssan et al. [49] reported that NMs, like ZnO, can improve animal health by promoting development and reducing diarrhea in piglets and dairy cows with recessive mastitis. Furthermore, Tsakmakidis et al. [50] study on FeO and Ag-NPs on ram sperm found that Ag-NPs demonstrated superior antibacterial activity and cytotoxicity, indicating potential for sperm therapy. Accordingly, Kot et al. [51] explored the effectiveness of metal NPs (Ag-NPs, Cu-NPs, Au-NPs, Pt-NPs, and Fe-NPs) in combating digital dermatitis in cows caused by *Treponema* bacteria, with Ag-NPs and Cu-NPs showing the most biocidal effect. Interestingly, Au-NP-based diagnostics in veterinary science have significantly improved the detection of pathogens and toxins in poultry and cattle, including bacterial infections like anthrax and brucellosis, thereby enhancing the quality of veterinary care [52]. Moreover, quantum dots (QDs) are being used to study livestock gamete biology and reproductive challenges. These biocompatible, photo-stable NPs can provide either targeted or non-targeted imaging with higher signal intensity than organic fluorescent molecules [53]. Based on this, Chatterjee and coworkers [54] proposed QDs-NPs activated by light to create superoxides as a treatment for drug-resistant bacterial infections, reducing viability by seven times. Additionally, researchers have developed QDFM immunochromatography for biological and chemical detection, offering a fast, efficient, specific, high-sensitivity, and simple operation, making it a potential immunolabeling technology [109].

#### 3.1.3. Hybrid NMs

Researchers have developed antibacterial drugs using NMs like graphene and polymers as matrix materials for metal NMs (Au, Ag, ZnO, Cu, and TiO_2_), enhancing biosensing and cell death [110,111]. Accordingly, Kaushal et al. [55] developed PEG@GO-decorated hybrid antibody biosensors for fast, specific, and higher sensitivity detection of foodborne bacteria like *E. coli* and *Salmonella typhimurium*, enabling faster NIR illumination and visual detection. Ahghari and coworkers [56] study on the sustainable synthesis of silver iodate NPs and chitosan (chitosan-AgIO_3_) showed high bacterial eradication rates against *E. coli*, *Klebsiella pneumoniae*, *Staphylococcus saprophyticus*, *Pseudomonas aeruginosa*, and *S. aureus,* indicating the potential of this green, inexpensive, and effective antibacterial agent for biomedical and therapeutic applications. In addition, polymeric NPs offer advantages over lipid-based NPs, including structural integrity, stability, and controlled release capabilities for drug delivery [112]. A potential antibacterial agent for preventing biofilms and intracellular bacterial growth and membrane formation was recently produced by Qiu and colleagues [113] using phosphatidylcholine-chitosan hybrid NMs doped with gentamicin antibiotics. Cui and colleagues [57] found that tea tree oil and liposome-loaded chitosan electro-spun nanofilms effectively inhibited *Salmonella* in chicken meat, while also preserving the sensory properties of the chicken meat, demonstrating their antibacterial potential in livestock food production. In another instance, Cui et al. [114] developed a chitosan edible film with liposome-encapsulated phage, enhancing phage stability and exhibiting high antibacterial activity against *E. coli* O157:H7, making it a promising antibacterial packaging for beef preservation. Pabast and colleagues [115] developed a biodegradable coating of chitosan with nano-encapsulated *Satureja khuzestanica* essential oils (SKEO) to improve food quality and extend shelf-life. The coatings effectively retarded microbial growth, delayed SKEO release, and enhanced sensory attributes, making them a promising candidate for lamb meat shelf-life extension. Furthermore, Amjadi and his team [58] developed betanin nanoliposomes (G/CH NF/ZnO NPs/B NLPs) using gelatin, chitosan nanofiber, and ZnO-NPs in a bio-nanocomposite film for meat preservation. The film effectively inhibited bacterial growth, lipid oxidation, pH changes, and color changes in beef samples, demonstrating its potential for meat preservation. Additionally, Huang and colleagues [116] manufactured *chrysanthemum* essential oil encapsulated with chitosan and pectin, which reduced oil release and demonstrated sustained antibacterial activity against *Campylobacter jejuni* in broilers through liposomal delivery.

### 3.2. Nanomaterials for Antiviral Applications

#### 3.2.1. Organic NMs

Biomedicine is advancing with organic NPs like liposomes, dendrimers, polymer micelles, and carbon-based NPs, promising antiviral candidates due to their viricidal activity, drug carrier properties, selective administration, and regulated release [117]. Accordingly, Bhattacharya and colleagues [59] used membrane-derived vesicles from human corneal epithelial cells, Vero, and CHO cells to combat HSV-1. These liposomes have receptors and neutralizing particles, but limitations in their drug carrier delivery require further research for optimal evaluation and production. Moreover, polyamidoamine dendrimers, naturally antiviral, prevent virus proteins from spreading, invading, and growing. They have the potential to combat diseases like *H1N1*, *HIV*, *SARS*, and *Ebola* [118,119]. Kandeel et al. [60] studied cationic and anionic dendrimers against *MERS-CoV* in vivo. The study found that anionic dendrimers reduced *MERS-CoV* by 40%, while cationic dendrimers assassinated Vero cells. Polyanionic dendrimers can improve targeted antiviral drug delivery.

According to recent studies, alternative treatments like early innate responses and Toll-like receptor ligands have being explored to prevent viral diseases in poultry animals [120]. On this basis, Bavananthasivam et al. [61] found that encapsulating TLR ligands in PLGA-NPs enhances IFN-γ and IL-1β expression, promoting prolonged innate responses and systemic immune responses against *Marek’s disease virus* (*MDV*) in chickens. Additionally, Singh and coworkers [121] found that PLGA-NPs effectively combat *H9N2 virus* in chickens, with nonencapsulated formulations generating higher antibody and mucosal responses. Moreover, Dhakal and colleagues [122] documented a new drug delivery platform using mucoadhesive chitosan NPs. The inactivated *swine influenza A virus (SwIAV)* vaccine, encapsulated in chitosan NPs, elicited strong immune responses in pigs, reducing viral shedding and lung virus titers, suggesting it as an ideal pig vaccine. Accordingly, Renukaradhya and coworkers [62] demonstrated polyanhydride-NPs, encapsulated in killed SwIAV, being effective as a vaccine in pigs, promoting virus-specific lymphocyte proliferation, fever protection, and reduced viral antigens for pigs. Furthermore, Huang and his research group [123] illustrated that mannosylated gelatin NPs (MnG-NPs) with inactivated *Porcine Reproductive and Respiratory Syndrome* (*PRRSV)* in vitro induce T cell-mediated immunity, enhancing monocyte dendritic cell uptake, cytokine expression, and cell activation, making it a significant PRSV vaccine for piglets.

#### 3.2.2. Inorganic NMs

Remarkably, Innocenzi et al. [63] identified that graphene, fullerenes, and carbon dots are promising antiviral agents due to their unique physicochemical characteristics. Graphene oxide has a large surface area and excellent sorption properties, while carbon dots are suitable for viral therapies like *HSV-1*, *HIV*, and *RSV* due to their high aspect ratio and superior mechanical properties. Ag-NPs have been studied for their antiviral effects on several viral infections, *including respiratory syncytial virus* (*RSV*), *dengue virus* (*DENV*), *influenza*, *hepatitis* (*HSV-1*), *poliovirus* (*PV*), and *coronaviruses* (*CoV*) [64]. Fruitfully, Yoo and coworkers [124] fabricated a heating filter membrane (HFM) decorated with plasmonic Au-NPs to eliminate *H1N1pdm09 virus* infectivity. The HFM reduced virus titers by over 99.9% in 10 min, and *SARS-CoV-2 virus* infectivity by 99% using the photothermal method. This meth by utilizing localized surface plasmon resonance, effectively inactivated the virus, making it suitable for air quality control, viral particle capture, and qRT-PCR genetic information extraction.

Additionally, inorganic NPs are widely used as antiviral agents in domestic animals [65]. Dung and his colleagues [66] found that Ag-NPs can effectively combat *African swine fever virus* (*ASFV*) in piglets, thereby reducing viral contamination in pig houses, indicating their potential as a disinfectant. Recently, Zeedan and colleagues [67] reported the biosynthesis of ZnO-NPs and Ag-NPs as antiviral agents against *bovine herpesvirus-1 (BoHV-1*) in cattl, demonstrating safety in Madin-Darby canine kidney cell culture and experimental animals with minimal cytotoxicity levels. Interestingly, Bai et al. [68] manufactured hollow mesoporous silica-NPs to induce persistent humoral immunity against foot and mouth disease virus-like particles in guinea pigs, enhancing T-lymphocyte proliferation and IFN-γ production, making them a promising nano-adjuvant for vaccines. On the other hand, Fawzy and colleagues [69] found that Au-NPs conjugated with *foot and mouth disease virus* (*FMDV*) capsid protein VP1 increased antibody production, IFN-γ production, and macrophage activity in guinea pigs.

#### 3.2.3. Hybrid NMs

Currently, hybrid NMs are progressively used in antiviral approaches due to their integration of antiviral substances, physical barriers, and photothermal or photocatalytic activity [125]. Recently, Ghaffari and his research team [126] investigated that ZnO-NPs with PEGylated coatings effectively inhibited H1N1 by decreasing MDCK-SIAT cell toxicity and improving antiviral activity. PEGylated ZnO-NPs showed 94.6% viral inhibition rates and decreased fluorescence emission intensity. In an additional study, Hodek et al. [127] fabricated a hybrid surface protection of Ag, Cu, and Zn on transparent glass or polymethylmethacrylate (PMMA) plates to combat viral transmission. The coating reduced HIV-1 titers by 99.5–100% after 20 min, while PMMA plates showed 75–100% and 98–100% inactivation after 120 min. The coating targets enveloped viruses, including *SARS-CoV-2*, and is sterile, safe for Vero and HeLa cells, and minimally cytotoxic. Interestingly, NMs provide targeted antiviral drug delivery with enhanced stability, controlled release, multifunctionality, and biological barriers, enhancing treatment outcomes. Recently, Smith and colleagues [128] developed hybrid poloxamer–lipid NPs to improve antiretroviral lamivudine delivery against HIV-1. M23TC, a phosphoramidite pronucleotide, improved the intracellular delivery and antiretroviral and pharmacokinetic profiles in MDM and CD4+ cells. Likewise, hybrid NMs improve antiviral delivery, clearance, and treatment strategies. In this regard, Abdel-Bar and his research team [129] employed lipid polymer hybrid NPs (LPH-NPs) in combination with piroxicam to administer azithromycin or niclosamide to counter the *Corona virus*. This system showed entrapment efficiencies, a dose-dependent cellular uptake, and enhanced antiviral efficacy.

Additionally, Zhou et al. [70] reported that GSH-ZnS NPs modified with zinc sulfide demonstrated significant antiviral activity against *PRRSV* in pigs, indicating potential for antiviral NM development and host restriction factor investigation. Interestingly, Zhou et al. [71] documented that MES-coated tellurium NPs (Te/BSA NPs) inhibited internalization, suppressing virus infection in *PRRSV* models and demonstrating higher antiviral activity against cattle and pigs. Recently, Du and colleagues [72] developed a method for fabricating virus-like particles using calcium phosphate-biomineralized core immunogen shell NPs, which were used to produce *FMDV* VLPs, suggesting it as an effective vaccine production method for cattle, sheep, and pigs. Likewise, Chen et al. [130] investigated the antiviral properties of graphene oxide sheets and GO sheets with Ag-NPs against *feline coronavirus* and infectious *bursal disease* virus in chickens.

### 3.3. Nanomaterials for Antifungal Applications

#### 3.3.1. Organic NMs

Organic NMs like micelles, dendrimers, liposomes, graphene, fullerene CNTs, and chitosan offer potential for antifungal therapy due to their large surface area, biocompatibility, targeted delivery, and biodegradability [131]. Leal and coworkers [73] confirmed that itraconazole encapsulated with liposomes had a synergistic effect against *Aspergillus* in vivo experiments. Adult female Wistar rats were exposed to *A. flavus*, and itraconazole encapsulated with liposomes showed higher antifungal activity. This drug could be used in clinical settings due to its cost-effectiveness and low cytotoxicity. For another illustration, Helal and his colleagues [132] highlighted the use of organic NMs against fungus-resistant strains and loaded antifungal drugs like nystatin and fluconazole. They found that the biological conjugation and encapsulation of NMs with drugs reduces the toxicity risk and offers promising antifungal therapy.

In addition, organic NPs such as polymeric NPs are being explored as potential antimicrobial drug delivery agents due to their efficient dissolving, entrapment, biocompatibility, low toxicity, and synergistic therapy capabilities in livestock [133]. On this basis, Maldonado et al. [74] proposed synthetic polymeric NPs and rapamycin, which can induce immune tolerance against Streptomyces hygroscopicus, potentially treating allergies, autoimmune diseases, and preventing antidrug antibodies in animal husbandry. Recently, the therapeutic potential of liposomal amphotericin B against *A. fumigatus*-induced pulmonary mycotic infections in livestock was reported by Siopi and coworkers [75]. Yet, Ahmed et al. [134] found that chitosan NPs effectively inhibited the growth of fungal-like oomycetes *Aphanomyces invadans* and *Saprolegnia parasitica* in fish, with the strongest concentration inhibiting 90 % of visible mycelial growth.

#### 3.3.2. Inorganic NMs

Inorganic NMs with a green synthesis approach have antibacterial, antifungal, and antioxidant properties. Metal and metal oxide NMs exhibit potential antifungal activity against Candida, Aspergillus, and dermatophytes. Amin et al. [135] prepared copper oxide (CuO) using *Aerva javanica* leaf extract to combat fungal infections. In vitro studies of CuO-NPs coupled with amphotericin B showed a higher MIC concentration (160 μg/mL), broad-spectrum activity, minimal toxicity, and a cost-effective approach. Inorganic NMs are also useful for antifungal therapy due to their increased solubility, stability, regulated release, and targeted administration to the infection site [136]. Significantly, these NMs have the potential to overcome the drawbacks of traditional antifungal medicines, including inadequate absorption and resistance to drugs. Recently, Gignone and his research team [76] incorporated clotrimazole into mesoporous silica using theoretical and analytic strategies, evaluating drug behavior through drug adsorption simulation and identifying high-loading-capacity configurations.

On the other hand, Hassan et al. [137] demonstrated that metal NPs like Fe, Zn, Ag, and Se have antimicrobial and antifungal properties, inhibiting mold growth and preventing mycotoxin production, and protecting against aflatoxins and mycotoxins in animals. Recently, Tawab and colleagues [77] demonstrated the antifungal effect of Fe_2_O_3_ and Fe_3_O_4_ NPs on Aspergillus flavus, isolated from broiler feed. These NPs were synthesized using the co-precipitate method, having a potent antifungal effect. In another study, Nabawy and his team [78] reported that higher concentrations of ZnO and Fe_2_O_3_ NPs inhibited *A. flavus* strains and decreased aflatoxin B1 production in cattle diseases, compared to commercial feed additives. Additionally, Alagawany and colleagues [138] reported that giving Japanese quail Se-NPs improved their growth, blood-related factors, corpse features, state of antioxidant immunity, and gastrointestinal flora, reducing their consumption of feed, as well as having antifungal activities.

#### 3.3.3. Hybrid NMs

Metal and metal oxide NMs such as Ag, Au, or ZnO can be combined with organic molecules, polymers, or carbon-based materials to create hybrid NMs for antifungal activities [139]. For instance, Reda et al. [79] used a sol–gel technique to create calcium-doped zinc oxide ceramic NPs (ZnO-CaO) for combating *Candida auris*. The ceramics showed better bioactivity and effectiveness in combating multidrug-resistant *C. auris*, as they release Zn^2+^, causing oxidative stress and DNA replication and ultimately killing the target microbe. Similarly, Hamad et al. [80] developed thiolated PEGylated cholesterol and PEG-SH nanocomplexes with Au nanorods in a poloxamer 407 hydrogel with fluconazole. These nanocomplexes reduced fungal proliferation (*C. albicans*) and improved cargo delivery by 14-fold, with minimal cytotoxicity towards human dermal fibroblasts. Also, Hernandez and his coworkers [81] developed titanium-doped copper dioxide/copper iodide (TiO_2_-Cu^2+^/Cul) composite NMs using the sol–gel and co-precipitation methods, with minimal inhibitory and fungicidal concentrations for *Candida parapsilosis* and *Aspergillus niger* making them cost efficient, and facile for the environment for biomedicine and environmental remediation. More specifically, hybrid NMs effectively disintegrate fungal cell membranes due to their high surface area, reducing proliferation and improving antifungal activity [140]. In this regard, Mohaptara and colleagues [141] prepared a green Ag-ZnO nanocomposite against *Schizosaccharomyces pombe*, reducing cell proliferation with minimal cytotoxicity, indicating potential antifungal activity in biomedicine and healthcare settings.

In addition, Masry and colleagues [142] found that nanobiotechnological applications in mycotoxicology are promising due to their size-dependent properties. They demonstrated that metal nanocomposites (Fe_3_O_4_/CuO/ZnO) can counteract ochratoxin residues in broilers by decreasing body weight, immunological responses, and oxidative stress, while enhancing kidney function. In another instance, Arias et al. [82] developed a miconazole nanocarrier using iron oxide NPs and chitosan, which demonstrated superior antifungal activity against *C. albicans* and *Candida glabrata* biofilms in veterinary applications, reducing CFU and metabolism and preventing external magnetic field effects. Interestingly, Atef et al. [143] found that ZnO-NPs and cinnamon oils effectively inhibited fungal growth in cattle mastitis, demonstrating a synergistic effect on the significant inhibition of fungal growth. Kalinska et al. [83] found that Ag-NPs, when combined with Cu-NPs, demonstrated strong antifungal activity against Candida albicans in dairy cows and goats; notably, Ag-NPs showed stronger activity than the Ag-Cu complex.

### 3.4. Nanomaterials for Antiparasitic Applications

#### 3.4.1. Organic NMs

Recently, standard treatments for parasite infections are facing resistance and poor functionality, prompting the development of organic nanomedicines as potential antiparasitic therapies. These materials reduce drug dosage and cytotoxicity and improve pharmacological potency [144]. In this regard, Moles and colleagues [84] developed an immunoliposome with antibodies targeting RBC surface protein glycophorin A, targeting naive and Plasmodium-infected RBCs. The liposomes loaded with chloroquinoline effectively transferred the drugs, inhibiting parasite growth. Furthermore, combination therapy encapsulates antiparasitic medications with immune modulators, improving treatment outcomes and enhancing bioavailability and therapeutic value [145]. Accordingly, Moles and his research group [85] confirmed immuno-PEG-liposomes for targeted drug delivery in a murine malaria model, efficiently encapsulating amphiphilic drugs like chloroquine and primaquine using a pH gradient. This method effectively inhibited parasite growth and improved drug activity after 15 min of exposure. Sawicka et al. [86] documented that liposome-based vaccines have strong immune responses against parasitic pathogens like *Toxoplasma gondii*. This reported that the intramuscular injection of MIC3 plasmids induced a significant and effective immune response against *T. gondii*, increasing serum levels of IgG2 and IgG1. Additionally, Zhang and his research group [87] reported a new approach to anti-coccidiosis drug formulation that involves using 3-carboxyphenylboronic acid-modified chitosan conjugates and diclazuril for site-specific drug release in chicken intestinal tracts. In another instance, a self-nanoemulsifying system (SNEDDS) has been developed to improve the solubilization capacity of buparvaquone (BPQ), a veterinary drug, for treating visceral leishmaniasis. The system, adsorbable on chitosan polymers, has shown enhanced oral bioavailability and potent in vitro efficacy in inhibiting parasite replication in the spleen and liver [88].

#### 3.4.2. Inorganic NMs

Moreover, artemisinin-based combination therapy (ACT) effectively treats mild malaria by targeting molecular markers and studying resistance genetics for improved results [146]. On this basis, Foko et al. [89] optimized and characterized the green synthesis of Ag-NPs using *A. cordifolia* leaves for potential medical uses. These polycrystalline, stable spheres showed strong antiplasmodial action against *P. falciparum* strains, making them safe for blood use. Green nanotechnology offers alternative malaria drug/insecticide development. Likewise, inorganic NPs aid in detecting and diagnosing parasitic diseases by interacting with receptors or biomarkers [147]. Additionally, inorganic NMs including mesoporous silica, metals (Cu, Ag, and Au), and metal oxides (TiO_2_ and ZnO) are gaining attention for their improved therapeutic efficacy against parasites like malaria, leishmaniasis, and toxoplasmosis [90,91,92]. In this regard, Adeyemi et al. [93] found that Au, Ag, and platinum (Pt) NPs have promising anti-*Toxoplasma gondii* therapeutic activity. Au-NPs and Ag-NPs showed a 13-fold increase in parasite killing compared to host cells, while Pt-NPs showed a 75% reduction in parasite growth. Tsamesidis et al. [148] studied silica-based NPs (Si-NPs) for improved drug delivery against malaria and leishmania parasites. They found that Si-NPs reduced leishmania activity but increased resistance to certain antileishmanial drugs. Furthermore, Jahani and colleagues [94] manufactured Au-NPs with labeled antigen B that can detect antibodies against the hydatid cyst disease of domestic animals, which is caused by *Echinococcus granulosus*, making it a simple, cost-effective, and selective early detection method. Additionally, cattle and buffalo are susceptible to Toxocariasis due to the gastrointestinal worm *Toxocara vitulorum*. Mohamed and colleagues [149] investigated the anthelmintic effects of Ag-NPs on both male and female worms as a result of drug resistance. Changes in body structure and the possible intake of drugs were identified. Recently, Ag-NPs synthesized from Azadirachta indica showed potent anthelmintic properties against *Haemonchus contortus*, a common parasite of domestic animals [95]. Another study by Aydin and colleagues [96] demonstrated that ZnO and FeO-NPs have anthelmintic effects on *Toxocara vitulorum* in cattle; these NPs caused oxidative/nitrosative stress, leading to the increased mortality of protozoans in the host.

#### 3.4.3. Hybrid NMs

Significantly, hybrid NMs combine organic and inorganic components for enhanced antiparasitic activity, drug delivery, stability, and bioavailability, improving treatment outcomes and vaccine formulation [150]. Very recently, investigations have aimed to identify immunogenic sites and reduce autoimmune and allergic reactions for effective parasitic vaccines. Oxidoreductase is a promising target in the SDR family for *Toxoplasma gondii* prevention [151]. In this regard, Yu et al. [97] developed TgSDRO-pVAX1, a DNA vaccine combining SDR family oxidoreductase, chitosan NPs, and PLGA. The vaccine demonstrated Th1/Th2 immunity, a transformed antibody production, dendritic cell development, and CD4^+^ and CD8^+^ T cell development in immunized mice, and that photodynamic therapy offers an alternative for treating localized lesions. Additionally, Sepúlveda et al. [98] synthesized TiO_2_ doped with Zn using solution combustion and hypericin (HY) for enhanced photodynamic activity against cutaneous leishmaniasis. The nanocomposite showed the highest fluorescence intensity and in vivo effects on the parasite load.

Elfeky et al. [99] developed cellulose nanocrystal (CNC) and ZnO/CuO nanostructures using the sol–chemical and hydrolysis approaches. The CNC/ZnO/CuO nanostructures showed better larvicidal efficacy towards Anopheles stephensi linked to CNC and ZnO/CuO nanostructures. Additionally, Shehu and coworkers [100] documented the biosynthesis of ZnO-CuO nanoporous composites using gum arabic; this composite has been efficaciously employed to control *Culex quinquefasciatus*, a vector of filariasis. Furthermore, Yang and colleagues [152] developed a magnetic field controllable and disposable electrochemical immunosensor for the detection of clenbuterol in pork samples. These sensors use graphene sheets, Nafion film, and Fe_3_O_4_@Au-NPs coated with bovine serum albumin–CLB conjugates, is sensitive, rapid, low-sample-consumable, and disposable.

## 4. The Mechanism of Nanomaterials for Antipathogens

Pathogens like bacteria, viruses, fungi, and parasites pose a significant threat to living organisms by causing infectious diseases and malignancies. While drugs are used to combat these resistant pathogens, nanotechnology and NMs offer potential solutions due to their antimicrobial properties [153]. Nonetheless, NM’s antimicrobial mechanisms remain unclear; current theories suggest that those mechanisms may involve direct contact, intracellular localization, and oxidative stress. NMs properties are influenced by physical, chemical, and morphological characteristics, leading to distinct modes of action [154]. Several key antipathogenic mechanisms of NMs are briefly discussed below.

### 4.1. Mode of Action of Nanomaterials for Antibacterial Activity

#### 4.1.1. Disruption to the Cell Membranes

The cell wall and membrane play a crucial role in maintaining the stability of the substances in the bacteria and protect the bacteria from harm [155,156]. NPs with antibacterial properties can attach to the negatively charged cell membrane due to their positive charge when they come into contact with bacteria [157]. The integrity of the cell membrane is damaged, weakening the interaction between lipopolysaccharide layers on the outer membrane. As a result, most lipopolysaccharides and proteins are released from bacteria, enhancing cell permeability and affecting material exchange inside and outside the cell [158]. In addition, NPs can penetrate the bacterial outer wall and accumulate in their inner membrane, causing instability, damage, increased membrane permeability, cell contents leakage, and death. For example, Au-NPs can continuously release ions that adhere to cell walls and membranes, altering the membrane permeability and causing the destruction of the bacterial envelope. Au-NPs can also cause damage through electrostatic attraction with bacterial cell walls, resulting in cell wall rupture and bacterial death [159,160,161].

#### 4.1.2. Production of Reactive Oxygen Species (ROS)

ROS are partially reduced oxygen derivatives with a strong oxidation capacity, including superoxide anions (O_2_^−^), hydrogen peroxide (H_2_O_2_), hydroxyl radicals (•OH), and singlet oxygen (^1^O_2_) [162,163]. Maintaining ROS at an appropriate level positively affects cells [164]. However, excessive ROS can have a negative effect and cause serious damage to bacteria [165]. The overproduction of ROS causes oxidative stress, which affects the structure and function of most biomolecules. For example, lipid peroxidation and protein oxidation are significantly increased [166,167], causing plasma membrane damage and cell apoptosis [168]. ROS mainly inactivate bacteria through two mechanisms: (i) the bacterial cell wall is destroyed, resulting in the leakage of cell contents or damage to the normal membrane transport system function, and the normal structure of related proteases is damaged to inactivate them [169]; (ii) ROS damage the sugar components and bases in genetic DNA, causing the double helix structure to be destroyed, causing normal bacterial proliferation and metabolism [170]. For example, Karunakaran and colleagues [171] found that positively charged 2H-MoS_2_ NPs can effectively attach to the surface of bacteria and stimulate more ROS production within bacterial cells. Qing et al. [172] demonstrated that Au-NPs can induce intracellular ROS production, potentially leading to bacterial death through protein aggregation and DNA destruction.

#### 4.1.3. Interaction with Cell Contents and Damage to DNA

**Figure 2:**
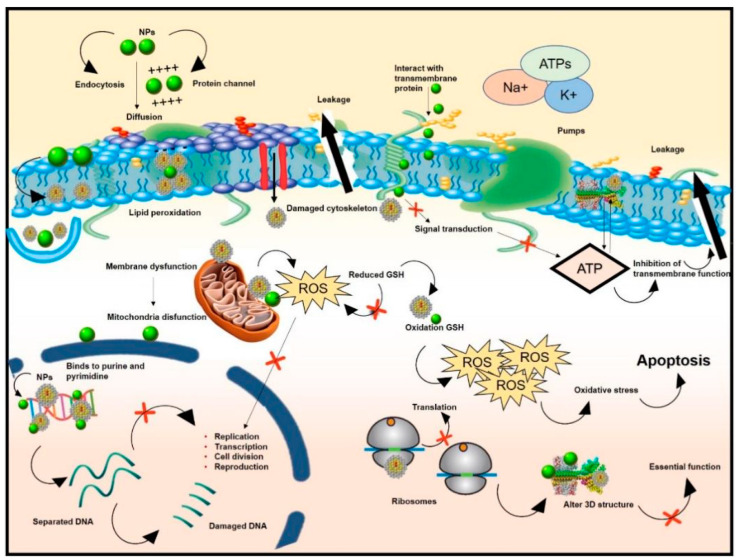
Diagrammatic representation of the antibacterial properties of nanoparticles.

Due to the small particle size of NPs, the antibacterial metal ions released in the solution, such as Ag^+^, Zn^2+^, etc., can enter and penetrate the cell, interacting with the cell contents such as proteins, enzymes, and genetic material to inactivate the cell [173]. Studies suggest that Ag^+^ reacts with protein sulfhydryl groups, inactivating proteins and inhibiting the activity of bacterial respiratory chain dehydrogenase [174,175,176]. NPs can inhibit bacterial replication and induce cell death by attaching to and binding to bacterial DNA, blocking DNA unwinding during transcription and preventing pathogen proliferation [177]. Lee and colleagues [178] confirmed that Au-NPs can induce DNA fragmentation and apoptotic-like cell death, independent of intracellular ROS. The key methods for various NM functions are summarized in Figure 2.

### 4.2. Mode of Action of Nanomaterials for Antiviral Activity

#### 4.2.1. NMs Directly Interact with the Viruses to Prevent Their Entry

The virus invades into the cell in three different stages: (i) the virus makes contact with the cell membrane, then enters the intracellular space, and subsequently releases the viral genome into the cell; (ii) the proliferation of the viral genome and its expression; (iii) the assembly of new viruses and their release into the extracellular space. NMs can directly influence virus replication, and they can also affect virus replication through immune responses [179]. Ag-NPs effectively combat viruses like *HIV-1* and monkeypox [180,181] by binding to gp120 through electrostatic interactions. Additionally, two disulfide links in the carboxyl half of the *HIV-1* gp120 glycoprotein are linked by Ag-NPs involved with sulfhydryl groups simultaneously, triggering protein denaturation by reducing disulfide bonds in the CD4 binding zone and inhibiting virus attachment to the host cell membrane [181]. Park and colleagues [182] synthesized a magnetic hybrid colloid loaded with Ag-NPs of varying sizes and found that Ag ions can bind to the sulfhydryl protein on the surface of the virus, thereby damaging the viral envelope and inhibiting the virus. Iron oxide NPs (Fe_2_O_3_ and Fe_3_O_4_) with glycine have been shown to reduce biotoxicity and inhibit the *H1N1 influenza virus*. The NMs with smaller diameters and higher surface areas demonstrated specific spatial resistance, effectively preventing virus attachment to host receptors [183]. The research conducted by Abo-Zeid [184] revealed that IO-NPs (Fe_2_O_3_ and Fe_3_O_4_) successfully interact with the *SARS-CoV-2* spike protein receptor binding domain and *HCV* glycoproteins. Notably, Fe_3_O_4_ forms a stable complex that disrupts the adsorption of the virus with host receptors.

#### 4.2.2. NMs Inhibit Viral Genome Replication

NMs can enter host cells, obstruct viral replication, and attach to viral genomes. Capping agents like polymers and surfactants enhance NM’s effectiveness, with capped Ag-NPs being highly efficient [185,186]. Ye and his research group [187] reported that graphene oxide (GO) inhibited virus replication against pseudorabies and porcine epidemic diarrhea in a cell culture. Negatively charged GO caused DNA damage and viral growth inhibition, while, when conjugated with nonionic PVP, it blocked viral infection. Additionally, Ghaffari and coworkers [126] demonstrated that surface-modified ZnO-NPs and PEGylated NPs effectively suppressed the *HSV-1* and *H1N1 influenza* virus replication at maximum non-toxic concentrations. The release of Zn^2+^ ions from an aqueous dissolution leads to cell apoptosis and potential oxidative stress and DNA damage in viruses.

#### 4.2.3. NMs Prevent Viruses Assembly and Release

Research has shown that metal ions can form chemical bonds with viral nucleic acids or proteins. This disrupts their structure or causes irreversible conformational changes in viral proteins, thereby achieving the goal of inhibiting viral replication. There are two plausible mechanisms that can account for the toxicity of Cu-NPs on viruses. The first mechanism is dissolution-independent, involving Cu^2+^capture. The second mechanism involves NPs instability, leading to the generation of large levels of Cu^2+^ [188]. Cu^2+^ ions can cause capsid disintegration, protein inactivation, and damage to the viral genome, effectively counteracting various viruses by impeding their entry into cells. Additionally, Cu-NPs can deactivate viral proteins in *HSV-1* through oxidation and genome destruction, releasing them into the extracellular space [189]. The virus attaches to the host cell, transcribes its genome, initiates replication, synthesizes mRNA and proteins, and aids in the reassembly of progeny virions [190].

#### 4.2.4. Activation of Immune System by Drugs That Can Hinder the Spread of Viruses

**Figure 3:**
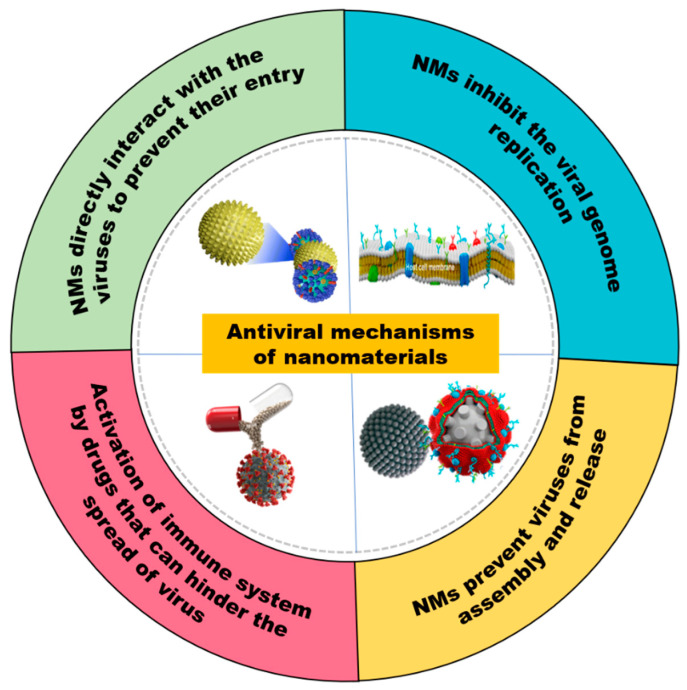
Schematic illustration showing the antiviral mechanism of nanomaterials.

Upon entering a cellular environment, viruses undergo essential processes like unpacking, replication, and translation, leading to the production of RNA/DNA molecules and proteins [191]. The host immune system can be stimulated by two mechanisms with respect to viral entrance into the host cell: (i) directly by NPs or (ii) by coating NPs to the drugs. Azharuddin et al. [192] documented that Au-NPs can trigger immunological responses, including humoral and cell-mediated responses, and produce M2e-specific IgG serum antibodies to prevent the spread of *influenza virus* by regulating cytokine generation and stimulating immune cells. On the other hand, NPs can stimulate the immune system when combined with drugs, inhibiting viral replication and their spread [193]. Figure 3 provides a summary of the key methods for the various NM antiviral function mechanisms.

With this regard, Dungdung et al. [194] utilized the ZnS quantum point as a drug carrier and loaded it with mycophenolic acid (MPA), an immunosuppressant against dengue virus. The study revealed a higher neutralization rate, enhancing the inhibitory effect and increasing the selective index by two-fold. Antiviral drugs can reduce virus infection rates, but the blood–brain barrier limits the drug scope. In this regard, Nair and coworkers [195] demonstrated the release of azidothymidine 5′-triphosphate, an anti-*human immunodeficiency virus* drug decorated with CoFe_2_O_4_@BaTiO_3_. This triggered release process is intrinsic, dissipation-free, and energy-efficient, achieving release at the intrinsic level without intermediate materials that help to prevent the viral spread into the host cell.

### 4.3. Mode of Action of NMs for Antifungal Activity

Metal-based NMs exhibit antifungal activity through three mechanisms: membrane rupture, interference with functions, and surface-dependent interactions with fungal cells, making them promising agents [196,197]. On this basis, Salah et al. [198] reported that Co-NPs can inhibit antifungal activity by preventing copper ion invasion and membrane degradation. However, they can also interfere with essential cellular functions, leading to cell death and affecting cell division and protein synthesis. In a different study, Matras and colleagues [199] found Ag-NPs potent antifungal properties in in vitro experiments on *F. avenaceum* and *F. equiseti* by disrupting the cell membrane structure, hindering budding activity, and preventing cytotoxicity. Munir and his research group [200] found that titanium ions permeate cell membranes and bind with DNA, while Cu-, Cr-, and Ni-doped TiO_2_ binds to fungal cells, enhancing its antifungal activity. Accordingly, Morsy and colleagues [201] discovered that CuO-NPs significantly impact broiler chickens growth, immune status, DNA status, and histological structures, with dose-dependent increases in malondialdehyde levels, Cu contents, and the DNA fragmentation percent.

### 4.4. Mode of Action of NMs for Antiparasitic Activity

NMs are currently being investigated for their potential antiparasitic effectiveness against various parasites by breaking down cell membranes, producing reactive oxygen species, transporting medication, stopping responses, regulating neurotransmission and enzyme activity, and activating the immune system [202]. In this regard, Villiers and his colleagues [203] found that chloroquine deposits in parasites digestive vacuoles prevented the detoxification of heme, leading to toxic hemozoin accumulation. Antiparasitic drugs can impact vital parasitic functions, hindering enzymes, blocking metabolic routes, depleting vital ions, and inhibiting immune defenses, ultimately causing parasite death. Khadragy and colleagues [204] found that biosynthesized Ag-NPs effectively combat *Leishmania* major infection, reducing cutaneous lesions and enhancing antioxidant enzyme activities in animals. In another instance, Torres et al. [205] documented metronidazole and riluzole to treat *Entamoeba histolytica*, causing DNA degradation, neurotransmitter interference, protozoan disintegration, nitric oxide generation, and parasitic cell death.

## 5. Issues and Challenges Need to Be Solved for the Utilizations of NMs as Antipathogens

**Figure 4:**
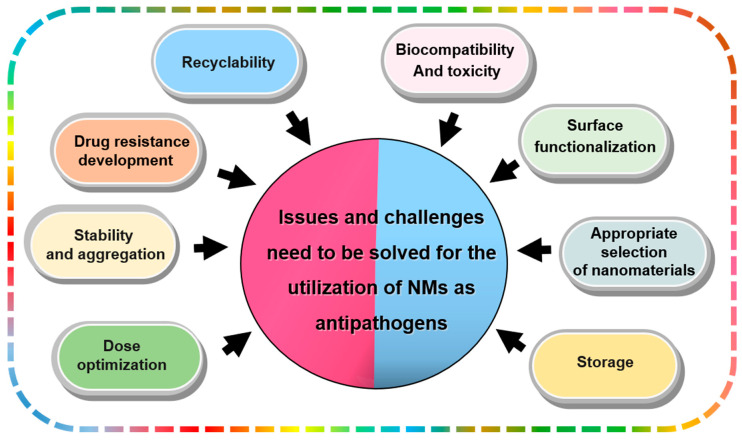
Issues and challenges regarding nanomaterials as antipathogens.

NMs are used to improve human and animal health through disease identification, prognosis, prevention, and treatment. Still, Researchers are exploring NM-based antipathogenic activity, which is crucial in medicine and agriculture. However, their use in biomedical applications faces challenges due to their adverse effects on living organisms, as shown in Figure 4.

### 5.1. Biocompatibility and Toxicity

Biocompatibility and toxicity are important factors to consider while using NMs in biomedical applications. Cytotoxicity is influenced by factors such as physicochemical properties, concentration, and exposure duration [206]. Genotoxicity, on the other hand, is affected by factors such as size, shape, surface charge, and composition, which can impact host cell and tissue interactions [207]. Ag-NPs could potentially cause DNA damage and chromosomal aberrations. Most specifically, NMs face challenges in blood contact, including protein adsorption, interference, and nanotoxicity, which can compromise their antimicrobial activity and cause adverse effects [208]. In vitro studies, using cell viability assays can identify NMs potential toxicity and safe concentrations, while in vivo studies evaluate their toxicological effects in complex biological systems using techniques like histopathology, immunohistochemistry, and biochemical analysis [209].

### 5.2. Appropriate Selection of Nanomaterials

Pathogen identification is a complex process that is influenced by various factors such as behaviors, strains, and genetic mutations. Understanding the target characteristics is crucial for developing effective NPs, avoiding harmful microorganisms [210]. Remarkably, an inappropriate selection of NPs can disrupt organelle distribution, affecting cellular processes like metabolism, protein synthesis, and waste disposal. Small NMs like Au-NPs and QDs have high stability and slow clearance rates, which increase the risk of long-term toxicity or bioaccumulation [211,212].

### 5.3. Surface Functionalization

The surface functionalization of NMs improves their stability, solubility, and antimicrobial properties, but precise control is challenging due to reaction conditions, surface impurities, and aggregation [213]. Functionalized NMs can disrupt living organisms, causing cytotoxicity, inflammation, and disrupting processes. For instance, Ag, Cu, TiO_2_, and ZnO-NPs possess antimicrobial properties but can cause toxic effects on living organisms, depending on characteristics including functional groups, dosage, and exposure duration [214,215].

### 5.4. Storage

Proper storage conditions are essential for NM’s stability and durability, as they are sensitive to environmental factors like temperature, moisture, and light. Improper storage can result in cytotoxicity, oxidative stress, inflammation, DNA damage, and potential health effects, including cancer development. For instance, metal and metal oxide-based NMs are prone to oxidation, while others are sensitive to air or humidity. Hence, understanding material-specific factors and storage conditions is crucial for long-term stability [216,217].

### 5.5. Dose Optimization

The dose is a crucial factor in antipathogenic applications, particularly, for antimicrobial treatments. It determines the efficacy and safety of therapeutic interventions. The optimal dosage is essential to balance antimicrobial activity and minimize negative effects, including toxicity, immune response, and bioaccumulation [218]. For instance, Ag-NPs, ZnO-NPs, TiO_2_-NPs, and CNTs have potential antimicrobial properties, but can cause toxicity, inflammation, organ dysfunction, and lung toxicity [219,220,221,222].

### 5.6. Stability and Aggregation

During antipathogenic activity, hostile hosts encounter stability challenges with nanoparticles, such as aggregation, precipitation, and dissolution. Aggregation reduces NPs effectiveness, while precipitation disrupts suspension stability due to factors like pH, temperature, or ionic strength, resulting in less effective particles, while the dissolution of NPs can compromise structural integrity, release toxic ions, and disrupt cellular processes.

### 5.7. Drug resistance Development

Drug resistance in pathogenic microorganisms poses significant challenges to traditional antimicrobial therapies. Novel antipathogenic agents face various obstacles, including evolution, biofilm resistance, cross-resistance, safety concerns, and environmental impact, so adaptive resistance is crucial in combating these challenges [223,224]. Multiple studies have revealed that bacteria can resist Ag-NPs through extracellular precipitation, destruction, or modification, similar to drug modification [225], while Cu-NPs induce antibiotic resistance by upregulating efflux pumps and membrane permeability [226]. Nevertheless, a lack of understanding in designing NMs hinders the design of rational strategies for drug resistance and antipathogenic activities, raises environmental concerns, and requires stricter regulations for regulatory approval and large-scale production [227,228].

### 5.8. Recyclability

NMs are effective in combating microbial infections, but they also face potential toxicity due to interactions with microorganisms, biological interactions, and aggregation. These interactions can lead to increased toxicity, potentially due to factors like size and shape. These interactions can result in the formation of a complex web of molecules that can interact with and bind to microorganisms, posing significant risks to the effectiveness of nanoparticles in treating microbial infections. Recyclability is crucial for utilizing NMs for antipathogenic activities and sustainable development and environmental protection. However, it can be challenging due to their small particle sizes, molecular penetration, aggregation tendencies, and time-consuming recycling processes [229,230,231].

## 6. Prospects

Antipathogenic activity based on NMs has immense potential for improving disease prevention, diagnosis, and therapy. Researchers are exploring novel NMs that can transport antimicrobial drugs directly to infected regions, improving localized infection treatment. These NMs can encapsulate drugs, protect them from degradation, and enhance their stability. Customization with ligands or antibodies can reduce dosage and improve drug delivery specificity and therapeutic benefits by boosting solubility, cellular absorption, or generating synergistic effects when coupled with drugs. In addition, nanofabrication techniques like 3D printing and bottom-up self-assembly offer promising methods for producing antipathogenic materials. These methods allow for precise control over their structure, composition, and characteristics, reducing resistance development, aiding in scalability, and enabling larger NM manufacturing for broader applications. In short, advancements in NMs and antipathogenic strategies have the potential to improve human and animal health and contribute to environmental remediation.

## 7. Conclusions

Nanotechnology and NMs offer customized tools for preventive and therapeutic purposes, addressing challenges in traditional antipathogen pathophysiology. These multifunctional NMs can overcome medicinal solubility, toxic exposures, uncontrolled pharmacokinetic issues, and biostability. Drug resistance occurs when high doses are insufficient to rapidly kill microbes, leading to the widespread distribution of untargeted drugs. However, nanocarriers offer molecular-level precision in targeting infected cells, allowing them to deliver multiple antigens to immune cells, which further allows for the development of better vaccines. In this respect, metal and metal oxide-based NMs and liposome-based NMs have been proven effective in preventing pathogen activity. However, production costs remain a significant concern, and clinicians must collaborate with the medical sector to adapt the technology for effective therapy.

## Figures and Tables

**Table 1:** Summary of nanomaterials for antimicrobial activities.

NMs	Nature	Antipathogens	Mode of Action	Therapeutic Outcome	Ref.
Polymeric micelles	Organic	*S. aureus*	Membrane lipases breakdown.	Multi-resistance drugs, biofilms	[41]
Chitosan HCL	Organic	Gram-negative and Gram-positive bacteria	Depolarizing the cell membrane.	Multi-resistance drug	[42]
Chitosan NPs	Organic	*E. coli*	Generate ROS production.	Antibacterial activity, meat preservation	[43]
Liposome	Organic	Gram-negative and Gram-positive bacteria	Break down cell membrane.	Antimicrobial activity, meat preservatives	[44]
Liposome	Organic	*Salmonella enterica*	Targeting viral cells; modified liposomes impair cellular processes.	Reduce microbial contaminants in poultry feed	[45]
Se-NPs	Inorganic	Gram-negative and Gram-positive bacteria	Increase ROS production.	Antimicrobial activity	[46]
Pd-NPs	Inorganic	*S. aureus*, *E. coli*	ROS induction via NIR.	Photothermal activity	[47]
ZnO-NPs	Inorganic	Gram-negative and Gram-positive bacteria	Induce ROS to disrupt essential proteins.	Multidrug-resistant bacteria in the poultry	[48,49]
Ag-NPs, Cu-NPs, Au-NPs, Pt-NPs, and Fe-NPs	Inorganic	*Treponema* bacteria	Oxidative stress damages cellular components.	Combating hoof disorders in cows	[50,51]
Au-NPs	Inorganic	*Bacillus anthracis*	Induce ROS to disrupt cell membrane.	Diagnostic marker in poultry and livestock	[52]
QDs	Inorganic	Gram positive and Gram-negative bacteria	The biochemical process is disrupted by damage to the plasma membrane and the cell wall.	Drug-resistant topical infections in livestock	[53,54]
PEG-GO-AuNPs	Hybrid	*E. coli*, *S. typhimurium*	Disrupt vital biomolecules by inducing ROS.	Biosensor, antibacterial agent	[55]
Chitosan-AgIO_3_	Hybrid	*P. aeruginosa*, *K. pneumoniae*, *S. saprophyticus*, *E. coli*, *S. aureus*	Oxidative stress damages cellular components.	Antibacterial activity	[56]
Liposome-loaded chitosan	Hybrid	*Salmonella* spp.	Activate reactive oxygen species, causing membrane breakdown when exposed to UV light.	Livestock food production	[57]
Betanin nanoliposomes (G/CH NF/ZnO NPs/B NLPs)	Hybrid	*E. coli*	Cellular components are damaged by oxidative stress.	Meat preservation, antibacterial effects	[58]
Liposomes	Organic	*HSV-1*	Modified liposomes target viral cells, disrupt cellular machinery.	Multi-resistance drug/biofilms	[59]
Dendrimers/PLL	Organic	*H1N1*, *HIV*, *SARS*, *Ebola*, *MERS-CoV*	Dendrimers interact with spike protein to inhibit DNA synthesis.	Antiviral drug delivery modulates the immune response	[60]
Polymeric lipid NPs	Organic	*MDV*	Modified polymeric lipids specifically target viral cells and interfere with biological processes.	Eliminate viral re-emergence	[61]
Polyanhydride-NPs	Organic	*SwIAV*	NPs enhance antigen adsorption, uptake, processing, maturation, immune response regulation, and are easily phagocytosed by APCs.	Lymphocyte proliferation, vaccines for pigs	[62]
Graphene, fullerenes, and CNTs	Inorganic	*HSV-1*, *HIV*, *RSV*	Electrostatic interactions with viral proteins to generate oxidative stress and immune responses.	Inhibiting viral replication, photothermal activity	[63]
Ag-NPs	Inorganic	*H1N1*, *H3N2*, *enterovirus 71*, *HSV-1/HSV-2*, *DENV*, *HIV poliovirus*	Plasma membrane rupturing and cell wall disruption, disturbs the biochemical process.	Eradicate viral replication	[64]
Cu, Ag, TiO_2_,graphene	Inorganic	*SARS-CoV-2*	Release toxic ions and ROS and UV-induced membrane destruction.	PDT, PTT, PPE, antiviral activity	[65]
Ag-NPs	Inorganic	*ASFV*	Damage to membranes due to free radicals and ROS.	Disinfectant	[66]
ZnO-NPs, Ag-NPs	Inorganic	*BoHV-1*	Cellular damage from oxidative stress	Antiviral agents	[67]
Mesoporous Si-NPs, Au-NPs	Inorganic	*FMDV*	ROS from ions disrupt homeostasis and permeate cells.	Vaccines	[68,69]
GSH-ZnS NPs,	Hybrid	*PRRSV*	Oxidative stress damages cellular components due to glycosylation and immunodominant decoy epitopes.	Antiviral activity	[70]
MES-coated tellurium NPs (Te/BSA NPs)	Hybrid	*PRRSV*	Te/BSA nanostars inhibit PRRSV proliferation and prophylactic effect.	Antiviral activity	[71]
Ca_3_(PO_4_)_2_ biomineralized core immunogen shell NPs	Hybrid	*FMDV*	The addition of polar amino acids to VLPs can enhance their stability in extreme environments, potentially improving their heat resistance.	Vaccines	[72]
Liposomes	Organic	*A. flavus*	Interact with the membrane, causing destabilization, cellular leakage.	Drug delivery, antifungal agent	[73]
Polymeric NPs	Organic	*Streptomyces hygroscopicus*	Antifungal activity involves cell membrane damage, causing cell death.	Drug delivery. treating allergies, autoimmune diseases	[74]
Liposomes	Organic	*A. fumigatus*	Liposome binding affinity for fungal cell walls ensuring stability and preventing toxicity.	Antimycotic infections, drug delivery	[75]
Si-NPs	Inorganic	*C. auris*	Ion’s release generates ROS disrupt homeostasis cause cell leakage.	Drug delivery, MDR	[76]
Fe_2_O_3_, Fe_3_O_4,_ ZnO NPs	Inorganic	*A. flavus*	ROS induces mitochondrial dysfunctional apoptosis.	Antifungal activity	[77,78]
ZnO-CaO	Hybrid	*C. auris*	Zn^2+^ disrupts zinc-mediated protein activity, generates oxidative stress.	MDR	[79]
Chol-PEG-SH, PEG-Fluc-GNR	Hybrid	*C. albicans*	Opsonization and phagocytosis inhibit DNA/RNA synthesis.	Drug delivery	[80]
TiO_2_-Cu^2^CuI	Hybrid	*A. Niger*, *C. parapsilosis*	Restrict enzyme function, release of Cu^2+^, alter NADPH generation.	MDR	[81]
Iron oxide and chitosan NPs	Hybrid	*Candida albicans* and *Candida glabrata*	ROS generation occurs when antifungal NMs attach to antifungal effect cells, elaborating O_2_ and metal ions.	Antifungal activity	[82]
Ag@Cu-NPs	Hybrid	*Candida albicans*	Release ions cause oxidative stress, cell wall damage, enzymatic activity inhibition.	Antifungal activity	[83]
Liposomes	Organic	*Plasmodium* spp.	Liposomes interact with ligands or antibodies and release encapsulated drugs.	Antiparasitic activity, drug delivery	[84]
PEG-liposomes	Organic	*P. falciparum*	Preventing immune system recognition and eliminating parasites through drug cellular uptake.	Conjugated therapy, drug delivery, MDR	[85]
Liposome	Organic	*Toxoplasma gondii*	Destabilizing membranes through acidic pH, disulfide bonding cleaving, and degradation.	Vaccines	[86]
Chitosan	Organic	*Eimeria* spp.	Chitosan destabilizes hydrophobic scaffolds in tertiary amines and degrades in response to intracellular environment.	Drug delivery	[87]
Chitosan	Organic	*Leishmania*	Chitosan destabilizes cellular membrane.	Drug delivery, antiparasitic activity	[88]
Ag-NPs	Inorganic	*P. falciparum*	Induce ROS causing cellular contents leakage.	Antiprotozoal activity	[89]
Au, Ag, Cu-NPs	Inorganic	*T. gondii*, malaria, leishmaniasis	Release ions, generate oxidative stress to kill parasites.	Biomarkers	[90,91,92]
Au, Ag, Pt NPs	Inorganic	*T. gondii*	Adsorption, permeation, and cytotoxicity of NPs with electrically charged substances.	Antiparasitic activity	[93]
Au-NPs	Inorganic	*Echinococcus granulosus*	AuNPs on hydatid cyst protoscoleces, assessing their effects on cell wall and caspase-3 activation.	Diagnostic marker	[94]
Ag-NPs	Inorganic	*Haemonchus contortus*, *Leishmania*	Free radicals induce oxidative stress.	Antiprotozoal activity	[95]
ZnO and FeO-NPs	Inorganic	*Toxocara vitulorum*	Oxidative stress and ROS generation increasing antioxidant enzyme activity.	Antiprotozoal activity	[96]
PLGA@chitosan	Hybrid	*T. gondii*	Acidic environment causes PLGA degradation, releasing drugs, and targeting parasites.	Vaccines	[97]
TiO_2_/Zn-HY	Hybrid	*L. amazonensis*	Oxidative stress inhibits DNA/RNA synthesis.	PDT, photosensitizer, and cutaneous leishmaniasis therapy	[98]
CNC/ZnO/CuO	Hybrid	*Anopheles stephensi*	Generation of hydroxyl ions and ROS leads to membrane disruption.	Photodegradation and larvicidal activities	[99]
ZnO-CuO nanocomposite	Hybrid	*Culex quinquefasciatus*	Generation of ROS antioxidant property of enzymes.	Antiprotozoal activity	[100]
